# Mechanistic insights into the role of traditional Chinese medicine in treating gastric cancer

**DOI:** 10.3389/fonc.2024.1443686

**Published:** 2025-01-21

**Authors:** Ziqiang Chen, Ting Yu, Yunhe Wang, Jiaxin Li, Bo Zhang, Liya Zhou

**Affiliations:** ^1^ College of Traditional Chinese Medicine, Changchun University of Chinese Medicine, Changchun, Jilin, China; ^2^ Department of Rheumatism, Third Affiliated Clinical Hospital to Changchun University of Chinese Medicine, Changchun, Jilin, China; ^3^ Department of Endocrinology, Metabolism and Gastroenterology, Third Affiliated Clinical Hospital to Changchun University of Chinese Medicine, Changchun, Jilin, China; ^4^ Changchun University of Chinese Medicine, Changchun, Jilin, China

**Keywords:** traditional Chinese medicine (TCM), gastric cancer, aerobic glycolysis, anti-angiogenesis, tumor microenvironment, immunomodulation, immunotherapy, molecular markers

## Abstract

Gastric cancer remains a leading cause of cancer-related mortality worldwide, with advanced stages presenting significant challenges due to metastasis and drug resistance. Traditional Chinese Medicine (TCM) offers a promising complementary approach characterized by holistic treatment principles and minimal side effects. This review comprehensively explores the multifaceted mechanisms by which TCM addresses gastric cancer. Specifically, we detail how TCM inhibits aerobic glycolysis by downregulating key glycolytic enzymes and metabolic pathways, thereby reducing the energy supply essential for cancer cell proliferation. We examine how TCM suppresses angiogenesis by targeting the vascular endothelial growth factor (VEGF) and cyclooxygenase-2 (COX-2) pathways, effectively starving tumors of nutrients and oxygen required for growth and metastasis. Furthermore, TCM modulates the immune microenvironment by enhancing the activity of effector immune cells such as CD4^+^ and CD8^+^ T cells and natural killer (NK) cells while reducing immunosuppressive cells like regulatory T cells (Tregs) and myeloid-derived suppressor cells (MDSCs). These actions collectively contribute to slowing tumor progression, inhibiting metastasis, and enhancing the body’s antitumor response. The insights presented underscore the significant potential of TCM as an integral component of comprehensive gastric cancer treatment strategies, highlighting avenues for future research and clinical application to improve patient outcomes.

## 1 Introduction

Gastric cancer is the fifth most common malignancy globally and ranks as the third leading cause of cancer-related mortality (1). Despite advancements in diagnosis and treatment, the prognosis for gastric cancer remains poor, particularly in advanced stages where the five-year survival rate is significantly reduced. In China, the situation is even more severe, with approximately 480,000 new cases and 370,000 deaths reported in 2022, accounting for 9.9% of all new cancer cases and 12.3% of cancer-related deaths (2).

Conventional treatments for gastric cancer, while effective for some patients, are often limited by toxicity, adverse side effects, and resistance, which significantly impact patient outcomes and quality of life. Characterized by natural compounds and fewer adverse effects, Chinese herbal medicine, in particular, uses natural substances tailored to individual needs to restore balance in the body (3). In the context of gastric cancer, specific herbs have been shown to directly target tumor growth, modulate immune responses, and alleviate side effects from conventional therapies, emphasizing both immediate therapeutic effects and long-term prevention (4).

The aim of this review is to critically explore how TCM addresses the biological mechanisms underlying gastric cancer. While conventional therapies remain essential, TCM’s unique, multi-targeted strategies hold potential to complement and enhance these approaches. This review will specifically focus on TCM mechanisms such as inhibiting aerobic glycolysis, suppressing angiogenesis, and modulating the immune microenvironment to achieve therapeutic benefits. By providing insights into these mechanisms, we aim to demonstrate TCM’s potential to improve treatment outcomes and suggest directions for future research and integration into modern cancer care.

## 2 Methodology

To ensure the relevance and quality of the reviewed studies, we conducted a comprehensive literature search across PubMed, Embase, and CNKI databases for articles published between 2017 and 2024. The search focused on studies examining the mechanisms of TCM in treating gastric cancer, including both preclinical and clinical research. Inclusion criteria were based on the relevance to gastric cancer and TCM, while studies unrelated to these topics, unpublished data, and non-peer-reviewed sources were excluded. This approach ensures that our review is grounded in high-quality, up-to-date evidence, enhancing the credibility and robustness of our analysis.

## 3 The role of traditional Chinese medicine in treating gastric cancer

### 3.1 TCM’s comprehensive regulation of gastric cancer cell behavior

has demonstrated multiple mechanisms of action in the treatment of gastric cancer, including inhibition of cancer cell proliferation, induction of apoptosis, antioxidant activity, and suppression of tumor metastasis (5). These mechanisms are interconnected and collectively work to inhibit tumorigenesis and cancer progression.

#### 3.1.1 Inhibition of cell proliferation

Uncontrolled proliferation is a hallmark of gastric cancer progression. TCM can inhibit cancer cell proliferation by regulating proteins involved in the cell cycle, such as Ki67, proliferating cell nuclear antigen (PCNA), and cyclin D1 (6). Ki67 and PCNA are key proteins associated with DNA replication and cell cycle regulation, while cyclin D1 plays a critical role in the transition from the G1 phase to the S phase of the cell cycle.

Studies have shown that certain TCM compounds can effectively regulate these proteins. For instance, curcumin, a polyphenol derived from turmeric (*Curcuma longa*), inhibits the expression of cyclin D1, reduces Ki67 expression through modulation of the NF-κB signaling pathway, and suppresses the proliferation of gastric cancer cells (7). Similarly, Panax notoginseng saponins have been found to reduce Ki67 and PCNA expression (8), thereby inhibiting cancer cell proliferation. Moreover, cynaroside, derived from *Alpinia katsumadai*, suppresses cyclin D1 expression and regulates the MET/AKT/mTOR signaling pathway (9), inhibiting both proliferation and migration of gastric cancer cells.

#### 3.1.2 Induction of apoptosis

Apoptosis, or programmed cell death, is a crucial mechanism for preventing uncontrolled cell growth and eliminating damaged cells. However, in gastric cancer cells, apoptosis is often suppressed, allowing tumor cells to survive and proliferate. TCM promotes apoptosis by regulating both intrinsic (mitochondrial) and extrinsic apoptotic pathways.

In the intrinsic (mitochondrial) pathway, apoptosis is controlled by a balance between pro-apoptotic proteins like Bax and anti-apoptotic proteins like Bcl-2 (10). In gastric cancer, Bcl-2 is often overexpressed, inhibiting apoptosis and promoting tumor progression. Studies show that TCM compounds such as Epiberberine can downregulate Bcl-2 expression and upregulate Bax, restoring apoptotic tendencies (11). Additionally, TCM such as Quercetin (12) and Helichrysetin C (13) has been found to increase the expression of key proteins in the intrinsic pathway, such as Caspase-3 and Caspase-9, leading to mitochondrial membrane disruption and cancer cell death.

In the extrinsic pathway, apoptosis involves the upregulation of death receptors on the cell surface (10). Ginsenoside Rg3, for instance, can activate Caspase-8 via the extrinsic pathway, enhancing apoptosis in cancer cells (14). Additionally, Resveratrol has been shown to upregulate DR4 and DR5 death receptors, sensitizing cells to TRAIL-induced apoptosis by activating Caspase-8 (15), leading to cancer cell death. This highlights the effectiveness of certain TCM compounds in modulating extrinsic apoptotic pathways. Di Na (16) et al. have shown that Astragalus memebranaceus can partially regulate the expression of Bcl-2 and Bax in HMrSV5 cells.

Overall, TCM induces apoptosis by modulating key proteins such as Bax, Bcl-2, caspase-3, and caspase-9 in both intrinsic and extrinsic pathways, thereby reducing tumor growth and demonstrating significant efficacy in gastric cancer treatment.

#### 3.1.3 Antioxidant activity

Oxidative stress, caused by an imbalance between reactive oxygen species (ROS) production and the body’s ability to neutralize them, plays a key role in the development of gastric cancer. Excessive ROS leads to DNA damage, promotes mutations, and supports cancer cell proliferation. Antioxidants present in TCM can eliminate ROS, reduce oxidative stress, and protect cells from oxidative damage.

Many TCM herbs are rich in antioxidant compounds such as flavonoids, polyphenols, and alkaloids, which effectively lower ROS levels and alleviate oxidative stress (17). For example, quercetin, a potent flavonoid, neutralizes ROS and activates endogenous antioxidant enzymes like superoxide dismutase (SOD) and glutathione peroxidase (GSH-Px), reducing oxidative stress in gastric cancer cells (18). This action inhibits proliferation and promotes apoptosis. Similarly, curcumin exhibits significant antioxidant activity by scavenging ROS and enhancing antioxidant enzyme activity, ultimately suppressing cancer cell proliferation and metastasis (19). Furthermore, berberine, an alkaloid extracted from plants such as *Coptis chinensis*, reduces ROS levels and DNA oxidative damage, effectively inhibiting the growth and metastasis of gastric cancer cells (20).

In conclusion, the antioxidant properties of TCM help to reduce oxidative stress by eliminating ROS and enhancing the body’s natural antioxidant defenses. These antioxidant effects not only protect cells from oxidative damage but also reduce cancer cell proliferation and may play a role in preventing metastasis.

#### 3.1.4 Inhibition of tumor metastasis

Metastasis, the spread of cancer cells from the primary tumor to distant organs, is a leading cause of death in gastric cancer. The process involves multiple steps, including cancer cell invasion, migration, and angiogenesis to support tumor growth at secondary sites. TCM has been shown to inhibit these processes, thereby reducing the risk of metastasis and improving patient outcomes.

A key mechanism in metastasis is the degradation of the extracellular matrix (ECM), which allows cancer cells to invade surrounding tissues and enter the bloodstream. This process is mediated by enzymes known as matrix metalloproteinases (MMPs), particularly MMP-2 and MMP-9, which are often overexpressed in gastric cancer. TCM compounds such as curcumin (21) and berberine (22) can downregulate MMP-2 and MMP-9 expression, inhibiting cancer cell invasion and reducing metastasis.

Additionally, TCM affects the epithelial-mesenchymal transition (EMT), a process in which epithelial cells lose their characteristics and gain mesenchymal traits, allowing them to become more mobile and invasive. For example, crocetin, an active compound from saffron (Crocus sativus), has been shown to downregulate EMT markers, inhibiting cell migration and invasion in gastric cancer cells (23). Resveratrol, a natural polyphenol, has been demonstrated to inhibit EMT by suppressing MALAT1, reducing the migration and invasion of gastric cancer cells (24). Dihydromyricetin, by regulating the JNK/MMP-2 pathway, reverses EMT and suppresses cancer cell migration and invasion (25).

In summary, TCM effectively inhibits gastric cancer invasion and metastasis by targeting multiple mechanisms such as MMP inhibition, EMT regulation, and the modulation of key signaling pathways, offering potential therapeutic strategies for gastric cancer treatment.

### 3.2 The mechanisms of TCM in regulating aerobic glycolysis in gastric cancer

AEG, also known as the Warburg effect, is a metabolic hallmark of gastric cancer cells. Unlike normal cells that rely on oxidative phosphorylation for energy production in the presence of oxygen (26), gastric cancer cells preferentially use glycolysis even under aerobic conditions. This metabolic shift supports rapid cell proliferation, helps evade apoptosis, and promotes drug resistance. Elevated levels of glycolytic enzymes and increased lactate production lead to an acidic microenvironment that favors tumor progression (27). Notably, Stage IV gastric cancer patients with lower pre-treatment lactate levels (≤5.0 mmol·L^-1^) have significantly longer survival times compared to those with higher lactate levels (>5.0 mmol·L^-1^) (28).

As gastric cancer advances, lactate concentration and the expression of key glycolytic enzymes increase, making lactate a critical biomarker of AEG activity (29). Lactate, a byproduct of AEG, facilitates tumor angiogenesis, disrupts the extracellular matrix, and creates an environment conducive to tumor growth (30). It also activates the pentose phosphate pathway, enhancing the production of antioxidants like NADPH and glutathione, which help cancer cells resist chemotherapy (31).

Key glycolytic enzymes and transport proteins involved in AEG include hexokinase 2 (HK2), lactate dehydrogenase A (LDHA), pyruvate kinase M2 (PKM2), phosphoglycerate kinase (PGK), phosphofructokinase (PFK), and pyruvate dehydrogenase kinase (PDK) (32). These enzymes facilitate glucose metabolism and lactate production. For example, HK2 initiates glycolysis by converting glucose to glucose-6-phosphate, accelerating tumor growth (33). PKM2 enhances AEG by converting phosphoenolpyruvate to pyruvate and interacting with regulatory proteins like hypoxia-inducible factor 1-alpha (HIF-1α), c-Myc, and STAT3 (34). AEG creates conditions that favor gastric cancer growth, proliferation, invasion, and metastasis (35). Although it produces less ATP than oxidative phosphorylation, AEG generates ATP more rapidly, meeting the energetic and biosynthetic demands of rapidly proliferating cancer cells (36).

**Figure 1:**
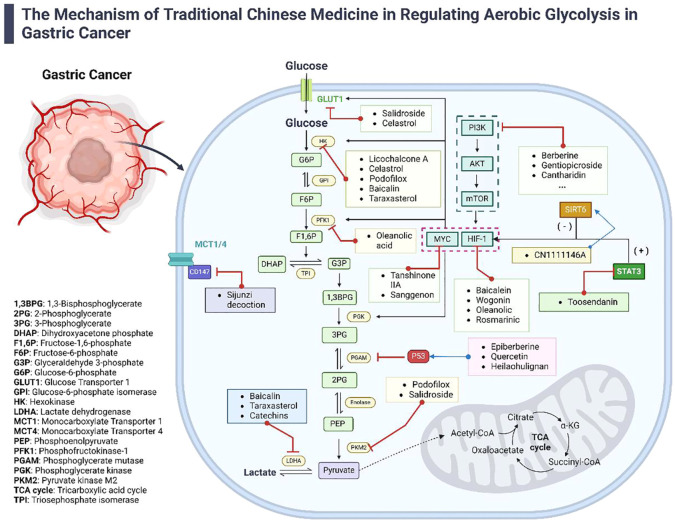
The Mechanisms of TCM in regulating aerobic glycolysis in gastric cancer. This diagram illustrates the diverse mechanisms by which TCM modulates aerobic glycolysis in the treatment of gastric cancer. It details how various active components from TCM influence AEG-related genes, proteins, enzymes, and signaling pathways. The figure highlights the synergistic effects of both TCM formulas and individual compounds in managing gastric cancer, providing a comprehensive view of TCM’s multifaceted approach to disrupting cancer metabolism.

Emerging research indicates that Traditional Chinese Medicine (TCM) targets the suppression of AEG by regulating related genes, proteins, enzymes, and metabolic pathways (refer to **Figure 1**). By inhibiting excessive glucose uptake by tumor cells and reducing lactate levels, TCM interventions focus on impeding tumor progression, potentially enhancing survival rates and improving the quality of life for patients (37).

#### 3.2.1 TCM regulation of AEG-related genes in gastric cancer

Cancer-related genes such as c-Myc are prevalent across various tumor tissues and critically regulate the expression of several genes involved in tumor cell metabolism at the post-transcriptional level (38). This regulation facilitates malignant transformation and proliferation. In gastric cancer, c-Myc collaborates with HIF-1α, a key protein in activating AEG (39), to enhance the expression of glycolytic enzymes like Glucose Transporters (GLUT), HK2, PFK, PGK, and LDHA, significantly elevating AEG levels in tumor cells. Studies have shown that specific TCM components, such as Tanshinone IIA (40) and Sanggenon C (41), inhibit the expression of c-Myc, along with signaling and transcription activators like STAT3 and HIF-1α in gastric cancer tissues, indirectly reducing AEG activity and thus curtailing tumor proliferation and metastasis. Additionally, Helichrysetin (HEL), a chalcone from *Alpinia katsumadai Hayata*, significantly inhibits the growth of gastric cancer cells by decreasing the expression and transcriptional activity of c-Myc (13), enhancing mitochondrial oxidative phosphorylation, and reducing glycolysis. HEL also targets the mTOR/p70S6K pathway, further contributing to its anti-tumor effects.

The tumor suppressor gene p53 is pivotal in regulating the cell cycle, differentiation, apoptosis, and DNA damage repair, maintaining genomic stability (42). Enhanced p53 expression can activate the TP53-induced glycolysis and apoptosis regulator (TIGAR), which lowers the levels of fructose-2,6-bisphosphate (F-2,6-P2), a promoter of glycolysis, thus inhibiting AEG (43). However, p53 is often mutated, underexpressed, or lost in tumor cells, leading to a metabolic switch from mitochondrial respiration to AEG. TCM compounds such as Epiberberine (44), which promotes p53 accumulation and induces G2/M cell cycle arrest and apoptosis through the Bcl-2/BAX/Caspase axis; Quercetin (45), which induces apoptosis by increasing ROS production, decreasing mitochondrial membrane potential, and modulating apoptotic proteins and genes; and Heilaohulignan C (46), which induces apoptosis via caspase and cytochrome C pathways and modulates p53, Bax, cleaved Caspase-3, and Bcl-2 levels, have all been demonstrated to increase p53 expression in gastric cancer tissues and cells, inhibit AEG, suppress gastric cancer cell proliferation, and promote apoptosis.

Given the extensive roles of c-Myc and p53 in tumor progression, and the limited research on how TCM interventions affect their influence on AEG, exploring their specific mechanisms of action is crucial for effectively inhibiting both AEG and gastric cancer progression.

#### 3.2.2 TCM regulation of non-coding RNAs in gastric cancer

Recent advances have spotlighted the role of non-coding RNAs (ncRNAs) in regulating various cellular functions in gastric cancer, including proliferation, apoptosis, and glucose metabolism (47, 48). With the aid of advanced technologies like microarray gene chips and PCR, researchers have identified abnormal expressions of long non-coding RNAs (lncRNAs), microRNAs (miRNAs), and circular RNAs (circRNAs) in gastric cancer (49), presenting new biomarkers for the disease’s development, diagnosis, and treatment.

*LncRNAs*, exceeding 200 nucleotides in length, play significant roles by influencing gastric cancer-related signaling pathways and the expression of oncogenes and tumor suppressor genes through the competitive endogenous RNA (ceRNA) mechanism, thereby impacting AEG in gastric cancer cells (50). For instance, the overexpressed lncRNA AK023391 in gastric cancer tissues has been identified as an independent prognostic factor. It enhances cancer cell proliferation and invasion by activating the PI3K/Akt pathway (51). TCM-derived Matrine, from *Sophora flavescens*, suppresses AK023391 levels in human gastric adenocarcinoma cells, thereby inhibiting their AEG and proliferation (51). Similarly, other lncRNAs like LOC101929709 (52) modulate the same pathway, influencing AEG in gastric cancer cells.

*MiRNAs*, consisting of 19 to 25 nucleotides, negatively regulate gene expression at the post-transcriptional level by binding to complementary sites on the 3’-untranslated regions (3’-UTR) of target mRNAs (53). Certain miRNAs like miR-320 (54) and miR-124 (55) regulate AEG by targeting the Krüppel-like factor (KLF)/HIF-1α pathway and the glycolytic enzyme PKM2, respectively.A study by Hanyu Zhang (56) et al: miR-200a, miR-559, and miR-1236 were negatively associated with CD86, CD81, and CD160, respectively, in almost all types of gastrointestinal cancers, which were further verified in the *in vitro* studies by transfecting microRNA mimics in gastric cancer, colon cancer, pancreatic, and esophageal cell lines. Additionally, tanshinone IIA impacts miR-124, decreasing PKM2 activity and thus the proliferative capacity of gastric cancer cells (49).

*CircRNAs*, arising from precursor RNAs through alternative splicing, long non-coding RNAs (lncRNAs) play an important role in the epigenetic regulation of cancer cells and regulate tumor progression by affecting chromatin modifications, gene transcription, translation, and sponge to miRNAs (57). This function indirectly controls gene transcription and protein expression, including those related to AEG. For example, circRNA-0009910, overexpressed in gastric cancer tissues, enhances cell proliferation, migration, and invasion (58). Total flavonoids from *Herba Patriniae* downregulate its expression, reduce lactate content in gastric cancer tissues, diminish cell proliferation and AEG levels, and enhance apoptosis (59). And, Fagen Li (60) et al. showed that Astragaloside IV (AS-IV) inhibited the development of GC through circDLST/miR-489-3p/EIF4A1 axis.

Given the substantial roles of ncRNAs in tumor progression and the nascent state of research on TCM interventions affecting their regulation of AEG, it is imperative to delve deeper into this area. Exploring how TCM influences ncRNAs that affect AEG is essential for elucidating micro-molecular mechanisms and could unlock new pathways for managing gastric cancer through unique molecular interactions offered by TCM.

#### 3.2.3 TCM regulation of key proteins in AEG in gastric cancer

##### 3.2.3.1 Key proteins initiating AEG

In gastric cancer, various proteins critically regulate the initiation and progression of AEG. Among these, HIF-1α, a 120 kDa protein comprising 826 amino acids (61), primarily regulates the body’s response to hypoxia. In the tumor microenvironment, however, HIF-1α is pivotal in reprogramming glucose metabolism. Its activation is influenced by multiple factors such as hypoxia, activation of signaling pathways, and cancer-related genes, collectively enhancing its expression (62).

Once activated, HIF-1α binds to the hypoxia response elements (HRE) in the promoter regions of genes encoding key glycolytic enzymes and transport proteins, thereby stimulating their activation and expression (63). The extrusion of lactate, the end product of AEG, further stabilizes HIF-1α expression, creating a positive feedback loop that perpetuates AEG (64). Therefore, targeting HIF-1α’s transcription, translation, and expression becomes a vital strategy for controlling AEG and halting gastric cancer progression.

Several TCM components, such as Baicalein (65), Wogonin (66), Oleanolic acid (67), and Rosmarinic acid (68), can effectively intervene in the transcription and expression of HIF-1α mRNA and protein. These interventions reduce glucose uptake by gastric cancer cells, decrease extracellular lactate levels, and inhibit the activation of downstream glycolytic enzymes. Moreover, some agents also impact the expression of transcription factors and proteins related to EMT and apoptosis, such as Snail, E-cadherin, Bax, and Bcl-2, further impeding gastric cancer progression.

Deacetylase Sirtuin family member 6 (SIRT6) exhibits anti-tumor properties and plays a critical role in regulating metabolic processes and enhancing AEG in tumor cells (69). In gastric cancer tissues and cell lines, a low expression of SIRT6 leads to the upregulation of HIF-1α and the activation of various AEG enzymes, thereby promoting gastric cancer cell growth (70). This growth is further facilitated by the activation of STAT3, which is highly expressed in tumor tissues and regulates essential malignant biological processes such as cell growth, proliferation, and angiogenesis. By modulating the activity of HIF-1α and c-Myc, STAT3 significantly enhances AEG and proliferation in gastric cancer cells (71), underscoring its pivotal role in the SIRT6-regulated AEG pathway. For example, the TCM formula “CN1111146A” has been shown to upregulate the expression of SIRT6, inhibit the activation of HIF-1α and key AEG enzymes, and delay the progression of gastric mucosal carcinogenesis in Atp4a-/- mice (72). Additionally, Toosendanin, an extract from Chuanlianzi, downregulates STAT3 expression, inhibiting HIF-1α activity and thus attenuating AEG in gastric cancer cells (73).

The cell cycle-related protein Rho effector molecule Rhotekin (RTKN) is often found highly expressed in gastric cancer tissues (74). Studies indicate that the alcoholic extract of Zuo Jin Wan can downregulate RTKN expression, reduce glucose uptake, decrease extracellular lactate content in gastric cancer cells, and inhibit cell proliferation (75). This suggests that a low expression of RTKN may inhibit AEG, thereby curbing the proliferation of gastric cancer cells. However, the specific mechanisms by which RTKN regulates AEG are not fully understood, necessitating further research to elucidate its role in the development of gastric cancer.

##### 3.2.3.2 Transport Proteins

The enhancement of AEG in gastric cancer cells is heavily reliant on the continuous uptake of glucose and the expulsion of lactate, a byproduct of this metabolic pathway (76). Transport proteins play a crucial role in this process; they work alongside glycolytic enzymes to facilitate not only AEG but also the transport of glucose and lactate across the cell membrane. GLUT1, in particular, is pivotal at the onset of glucose metabolism for transporting glucose from outside the cell into the cytoplasm. Its elevated expression in tumor tissues typically signifies increased cellular glucose uptake and enhanced AEG activity (77).

When significant amounts of lactate are produced during AEG, Monocarboxylate Transporters 1 and 4 (MCT1 and MCT4), along with their accessory protein CD147, are essential for transporting lactate from the intracellular environment to the outside of the cell (78). This increased activity in glucose uptake and lactate expulsion not only drives the AEG process in tumor cells but also contributes to the creation of an acidic extracellular environment. This acidic milieu is conducive to tumor progression and invasion, highlighting the critical role of transport proteins in the metabolic dynamics of cancer cells.

Inhibiting the expression of transport proteins that regulate the uptake and expulsion of glucose and lactate is a critical strategy for cutting off the essential “raw materials” needed for gastric cancer cell proliferation. This approach also ameliorates the acidic microenvironment conducive to cancer progression, effectively suppressing AEG enhancement and treating gastric cancer. For instance, the TCM formula Si-Jun-Zi Decoction has been shown to inhibit the expression of MCT1 and MCT4, and their accessory protein CD147, in rat gastric mucosa (79). This action reduces extracellular acidification levels, thereby delaying the progression of gastric mucosal carcinogenesis.

Additionally, specific TCM components such as Salidroside (80), Celastrol (81), and cardiac glycosides like Ouabain, Oleandrin, and Digoxin (82) have demonstrated efficacy in downregulating the activation of GLUT1. These agents reduce intracellular glucose levels, weakening the AEG process in gastric cancer cells. These findings highlight the potential of targeted interventions against transport proteins as a viable therapeutic approach in managing gastric cancer, leveraging the unique properties of TCM formulations and compounds.

#### 3.2.4 TCM regulation of key enzymes in AEG in gastric cancer

TCM targets the key enzymes to disrupt the enhanced glycolytic pathway favored by gastric cancer cells. Compounds such as Licochalcone A (83) and Celastrol (81) are known to inhibit HK2. Similarly, agents like Podofilox (84) and DT-13 (85) suppress both HK2 and PKM2, while Baicalin (65) and Taraxasterol (86) specifically target HK2 and LDHA. β-Asarone (87) inhibits PDK, and Oleanolic acid (67) suppresses PFK activity. Additionally, Catechins counteract the catalytic actions of LDHA by promoting ROS production (88). These interventions can significantly alter the metabolic profiling of gastric cancer cells, reducing their proliferation and invasiveness.

The primary role of AEG is to swiftly produce ATP, providing essential energy to tumor cells. Within the glycolytic pathway, only PKM2 and PGK are involved in this ATP generation process, directly impacting tumor cell growth and proliferation by influencing their energy supply. Clinical studies have shown that expressions of PKM2 and PGK are significantly elevated in gastric cancer tissues compared to adjacent non-cancerous tissues, correlating with TNM staging (89). This underscores the critical role of these enzymes in gastric cancer progression. TCM interventions such as modified Jianpi Yangzheng Decoction (mJPYZ) (90) and Salidroside (80) have been shown to inhibit PKM2 expression in gastric cancer cells, promoting apoptosis and reducing proliferation. However, no TCM interventions have yet been reported to specifically target PGK activity, highlighting a gap and the need for further research into TCM components that might regulate this enzyme.

#### 3.2.5 TCM regulation of AEG-related signaling pathways in gastric cancer

##### 3.2.5.1 TCM interventions on PI3K/Akt/mTOR pathway

The PI3K/Akt/mTOR signaling pathway, a central regulator of cell growth, metabolism, and survival, is frequently activated in gastric cancer (91) and precancerous lesions (92). This pathway influences the transcription and translation of key AEG initiators like HIF-1α, thereby affecting the initiation of AEG and the expression of related enzymes (93). Numerous studies confirm the efficacy of TCM components in targeting this pathway to inhibit AEG in gastric cancer cells. Schisandrin B liposomes (94) and Cantharidin (95) comprehensively inhibit the activation of the PI3K/Akt/mTOR pathway. These interventions prevent excessive proliferation of gastric cancer cells and various malignant phenotypes. For example, Schisandrin B liposomes downregulate the expression of HIF-1α and the epithelial-mesenchymal transition marker, N-cadherin. Compounds such as Berberine (96), Zi-yin-hua-tan recipe (97), mJPYZ (98), and Gentiopicroside (99) inhibit the expression of proteins in this pathway, impacting lactate content and glucose uptake, thus reducing AEG levels in gastric cancer cells.

##### 3.2.5.2 TCM interventions on Wnt/β-catenin signaling pathway

The Wnt/β-catenin pathway, frequently activated in gastric cancer, plays an essential role in tumorigenesis and energy metabolism. The finding that both PI3K/AKT/mTOR and ErbB pathways are involved in the development of gastrointestinal cancers and the confirmation of their association with m1A-regulated genes have provided direction for our research (91). For example, Berberine has been shown to inhibit the activation of the Wnt/β-catenin pathway in both the MGC803 and SGC7901 xenograft tumor models (100). This inhibition leads to a reduction in lactate production and lowers the expression of JNK, a protein that promotes the expression of glycolytic enzymes. Additionally, the presence of ROS in tumor tissues can initiate apoptosis and suppress YAP expression, which significantly impacts glucose metabolism in tumor cells (101). An instance of this is the Curcumin analog WZ35, which increases ROS levels in BGC-823 cells, thereby inhibiting the activation of the YAP/JNK pathway and consequently reducing levels of cellular AEG (102).

While interventions in the PI3K/Akt/mTOR pathway are well-documented, studies on other pathways in gastric cancer are limited. The tumor microenvironment in gastric cancer involves multiple dysfunctional signaling pathways contributing to sustained AEG activation. Given the multi-target, multi-level advantages of TCM, exploring herbs and formulas that can intervene in multiple proteins or pathways is beneficial for further suppressing AEG. This approach underscores the potential of TCM to provide a holistic treatment strategy that addresses the complex interplay of various signaling pathways involved in cancer metabolism.

### 3.3 TCM inhibits angiogenesis in gastric cancer

Angiogenesis, the formation of new blood vessels, is crucial for the growth and metastasis of gastric cancer. Tumors secrete various angiogenic factors, such as vascular endothelial growth factor (VEGF), to stimulate the development of new blood vessels (103), ensuring an adequate supply of oxygen and nutrients. Inhibiting angiogenesis can effectively starve tumors, limiting their growth and metastatic potential.

The VEGF family includes VEGF-A, VEGF-B, VEGF-C, VEGF-D, VEGF-E, and placental growth factor (PIGF), with receptors VEGFR-1, VEGFR-2, and VEGFR-3 (104). Among these, VEGFR-2 plays a critical role in tumor angiogenesis. Studies have shown that VEGF and VEGFR are highly expressed in gastric cancer tissues and are associated with the proliferation and invasion of gastric cancer cells (105). Comparative studies have found that VEGF-A, VEGF-C, and VEGF-D expression levels are higher in gastric cancer and precancerous lesions than in normal tissues, with the highest levels in gastric cancer tissues. Higher VEGF expression correlates with poorer patient prognosis.

Cyclooxygenase-2 (COX-2) is an inducible enzyme significantly overexpressed in various gastric cancer tissues and is associated with several carcinogenic processes, including tumor angiogenesis, inhibition of cancer cell apoptosis, and suppression of immune responses (106). Prostaglandins produced by COX-2 catalyze vascular dilation and increase blood flow within new blood vessels, which is essential for tumor angiogenesis (107).

Basic fibroblast growth factor (bFGF), also known as FGF-2, is vital for cell growth and proliferation (108). bFGF targets endothelial cells lining blood vessels, binding to specific receptors and initiating signaling events that promote cell proliferation, migration, and differentiation. Elevated levels of bFGF in tumors are associated with poor prognosis, highlighting bFGF as a critical target for anti-angiogenic therapy aimed at disrupting the tumor’s blood supply and inhibiting growth.

**Figure 2:**
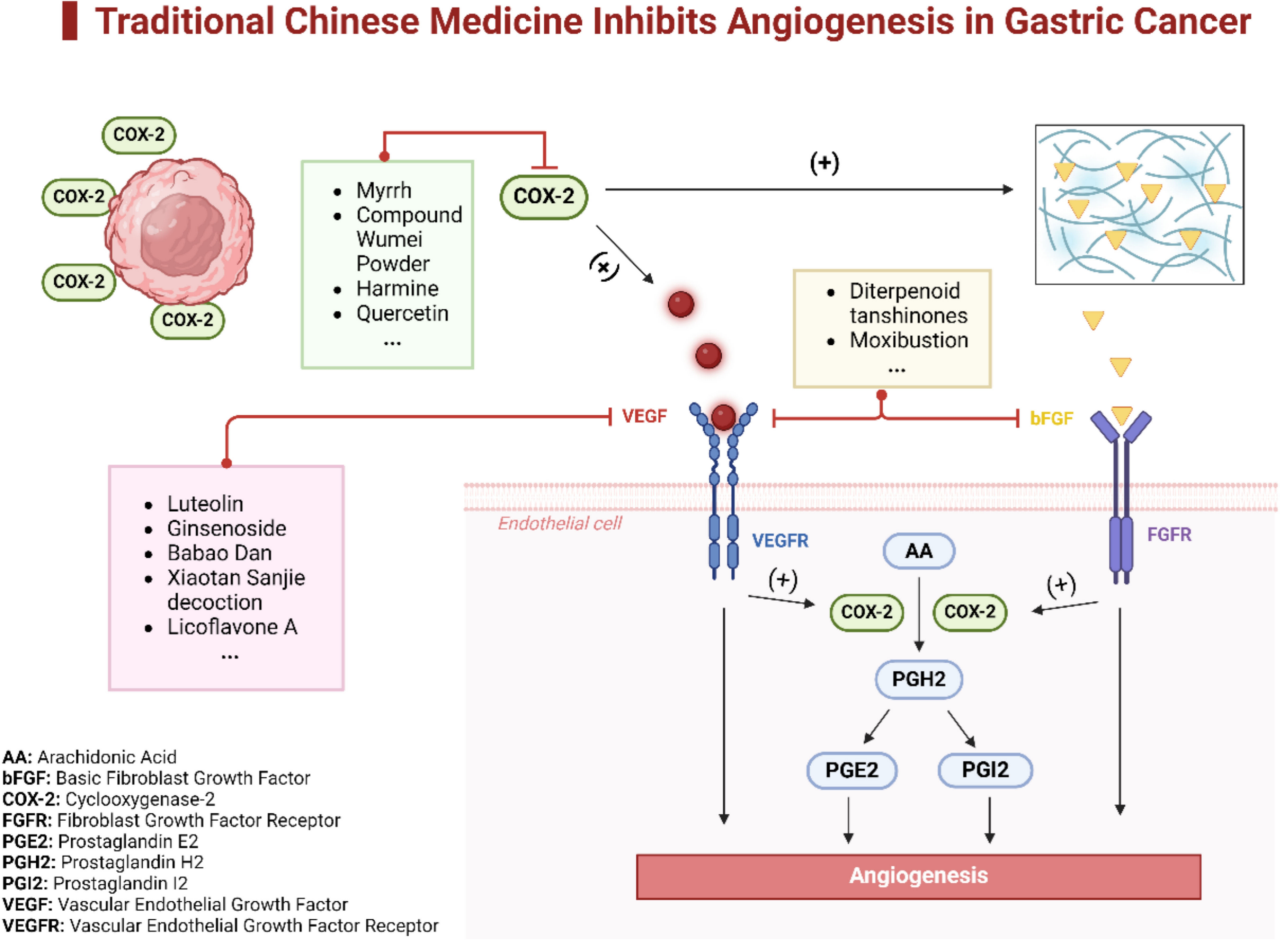
TCM Inhibits angiogenesis in gastric cancer. This diagram illustrates the multifaceted mechanisms through which TCM inhibits angiogenesis in gastric cancer. It highlights specific TCM components that suppress angiogenesis by different pathways. Some components downregulate the expression of COX-2, which in turn diminishes the activity of the VEGF pathway, thereby reducing angiogenesis. Other components directly target endothelial cells, interrupting the signaling pathways of both beg and VEGF, ultimately inhibiting tumor angiogenesis.

There is substantial evidence that TCM can significantly inhibit tumor angiogenesis. TCM interferes with tumor-induced angiogenic signaling by targeting VEGF, COX-2, and bFGF, thereby weakening the tumor’s ability to nourish and sustain itself (**Figure 2**).

#### 3.3.1 Inhibition of VEGF pathway

In the context of inhibiting angiogenesis in gastric cancer, various components of TCM have demonstrated effectiveness by modulating the VEGF signaling pathway. For example, Luteolin, extracted from an aromatic flowering plant, significantly inhibits VEGF protein expression involved in the Notch1/VEGF pathways, displaying profound antiangiogenic activity (109). Additionally, a study on a VEGFR-3 antibody-conjugated Ginsenoside Rg3 nanoemulsion (VRIN) highlighted its significant impact in reducing tumor growth and lymph-node metastasis. This was achieved by suppressing the expression of VEGF-C, VEGF-D, and VEGFR-3 in an orthotopic mouse model of human gastric cancer, underscoring TCM’s potential in treating metastatic gastric cancer (110). Babao Dan also plays a role by inhibiting growth and angiogenesis in gastric cancer within human umbilical vein endothelial cells (HUVECs). It regulates the VEGFA/VEGFR2 signaling pathway, effectively reducing cell viability, migration, and VEGFA levels in a concentration-dependent manner (111). Furthermore, the Xiaotan Sanjie decoction has been found to curb angiogenesis in gastric cancer by regulating IL-8 and downregulating components of the VEGF pathway. This affects tube formation, migration, and the expression of both protein and mRNA of VEGF-A and its receptors in co-cultured HUVECs and gastric cancer cells (112). Lastly, Licoflavone A, a natural flavonoid from Glycyrrhiza, has been demonstrated to inhibit gastric cancer proliferation, induce apoptosis, and reduce migration, invasion, and epithelial-mesenchymal transition by targeting VEGFR-2 and disrupting the PI3K/AKT and MEK/ERK pathways in both *in vitro* and *in vivo* settings (113).

#### 3.3.2 Inhibition of COX-2 pathway

Recent studies have underscored the efficacy of TCM components in suppressing angiogenesis in gastric cancer, primarily through the downregulation of COX-2 expression. For instance, myrrh has been shown to significantly inhibit the proliferation and migration of gastric cancer cells, and to induce apoptosis by downregulating COX-2 expression both *in vitro* and *in vivo (* 114). Compound Wumei Powder (CWP), a blend of equal parts Wumei (dried fruit of Prunus mume) and Wuweizi (dried fruit of Schisandra chinensis), has demonstrated its capacity to curb invasion and metastasis of gastric cancer cells through its impact on the Cox-2/PGE2-PI3K/AKT/GSK3β/β-catenin signaling pathway, both *in vitro* and *in vivo (* 115).

Additionally, the combined application of paclitaxel and harmine was found to synergistically inhibit cell proliferation, migration, and invasion in two human gastric cancer cell lines, SGC-7901 and MKN-45, by downregulating COX-2 and MMP-9, suggesting its potential as an effective therapeutic strategy for gastric cancer (116). Moreover, a study demonstrated that combining Quercetin with low-dose irinotecan/SN-38 in the AGS human gastric cancer cell line, both *in vitro* and *in vivo*, was as effective as high-dose SN-38 alone. This combination reduced cell viability, increased apoptosis, and downregulated β-catenin, COX-2, and angiogenesis markers, highlighting Quercetin’s potential to enhance the anticancer effects of irinotecan/SN-38 (117). These findings collectively underscore the potential of TCM components to target and disrupt key pathways involved in tumor angiogenesis and metastasis, affirming their role in developing novel therapeutic approaches for gastric cancer.

#### 3.3.3 Inhibition of bFGF pathway

Despite the proven efficacy of targeting growth factors like bFGF and VEGF in cancer treatment, research focusing specifically on bFGF in gastric cancer remains limited. However, notable studies have examined the impact of TCM components on this pathway. For instance, research on Diterpenoid Tanshinones (DT) from Salvia miltiorrhiza Bunge has shown that they can inhibit angiogenesis by affecting both the VEGF and bFGF pathways. Using network pharmacology to identify relevant targets and pathways, this study demonstrated that DT significantly reduces the proliferation and migration of gastric cancer cells and endothelial cells, decreases levels of VEGF and bFGF, and suppresses tumor growth and angiogenesis *in vivo*, likely via the PI3K/Akt/mTOR pathway (118). Another study evaluated the effects of moxibustion on tumor growth, metastasis, and body weight in rats with gastric tumors. It revealed notable improvements in body weight and reductions in tumor growth and metastasis compared to controls. This study also observed significant changes in the levels of key angiogenic factors, including reductions in both VEGF and bFGF levels in tumor tissues, highlighting the complex regulatory effects of moxibustion on angiogenesis and its potential as a therapeutic option in managing gastric cancer (119).

While significant advancements have been made in gastric cancer research, challenges remain, particularly in translating the anti-angiogenic effects of TCM from *in vitro* studies to clinical applications. Although TCM formulas are widely used and show promising anticancer effects, the mechanisms underlying these effects are not fully understood. Nevertheless, ongoing advancements in modern scientific techniques are expected to yield positive results, progressively elucidating the mechanisms by which TCM combats gastric cancer, including its role in inhibiting angiogenesis. This is expected to provide a more robust scientific basis for the clinical use of TCM treatments.

### 3.4 TCM’s regulation of the immune microenvironment

The immune microenvironment plays a crucial role in the progression of gastric cancer and the body’s ability to combat it. Gastric tumors create an immunosuppressive environment by recruiting regulatory T cells (Tregs), myeloid-derived suppressor cells (MDSCs), and tumor-associated macrophages (TAMs), which inhibit the antitumor activity of effector immune cells such as CD8^+^ T cells and natural killer (NK) cells. This suppression disrupts the body’s natural immune response against tumor cells, allowing the cancer to thrive. An imbalance in CD4^+^ T cell subsets, particularly a shift from Th1 to Th2 dominance, is often observed in the gastric cancer microenvironment. Th1 cells secrete cytokines like interferon-gamma (IFN-γ) and tumor necrosis factor-alpha (TNF-α), promoting cytotoxic T cell proliferation and tumor cell eradication. Conversely, Th2 cytokines inhibit Th1 cell proliferation, aiding immune evasion and further compromising the antitumor immune response (120).

CD8^+^ T cells and NK cells are crucial for targeting and eliminating tumor cells. However, their function is compromised in the immunosuppressive tumor microenvironment due to the inhibitory effects of Tregs, MDSCs, and other suppressive factors (121). Dendritic cells (DCs), essential for antigen presentation and activation of T cells, also suffer reduced infiltration and function in this environment, leading to insufficient T cell activation and aiding immune evasion (122). The accumulation of immunosuppressive cells like Tregs, TAMs with an M2-biased phenotype, and increased MDSCs enhances immune evasion and supports tumor progression (123). These cells release cytokines that further suppress effector immune cells and promote tumor growth and metastasis (124).

**Figure 3:**
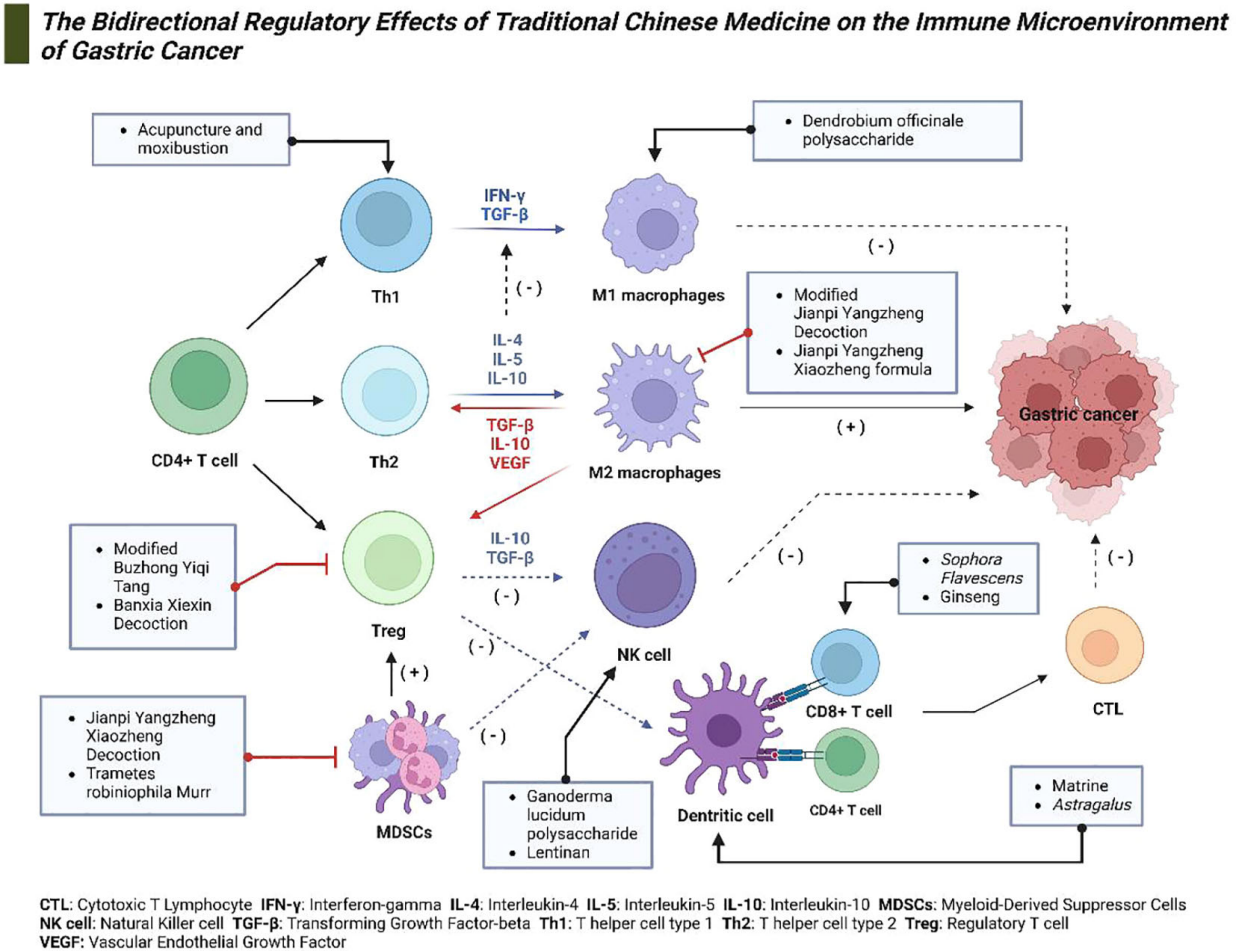
The bidirectional regulatory effects of TCM on the Immune Microenvironment of gastric cancer. This diagram depicts the mechanisms through which TCM modulates the immune microenvironment in gastric cancer. Various constituents of TCM target specific immune cells and cytokines, including Th1 and Th2 cells, regulatory T cells (Tregs), natural killer (NK) cells, dendritic cells, and macrophages. These interactions facilitate anticancer activities and modulate immune responses effectively.

Understanding these mechanisms is critical for developing effective treatments for gastric cancer. Targeting the immune microenvironment to enhance the body’s antitumor response is a promising therapeutic strategy. TCM offers distinctive advantages in this regard, as it can modulate the immune microenvironment bidirectionally. Extensive research indicates that TCM can strengthen immune function and enhance the cytotoxicity of immune cells while also reducing immunosuppression to prevent immune escape (125). This section will explore how TCM can enhance immune function and increase the killing capability of immune cells, as well as how it can reduce immunosuppression and prevent immune escape (**Figure 3**).

#### 3.4.1 Enhancing immune function and increasing the killing capability of immune cells

##### 3.4.1.1 Enhancing the anti-tumor ability of CD4^+^T and CD8^+^T cells

TCM formulations and compounds have shown potential in correcting Th1/Th2 imbalances and enhancing the cytotoxic capabilities of both CD4+/CD8+ T cells (5). Notably, techniques such as acupuncture and moxibustion have been shown to extend PFS and OS in advanced gastric cancer patients following second-line chemotherapy by reversing the Th1/Th2 shift and reducing inflammatory responses (126). Additionally, a study (127) investigating the integration of Compound Sophora Flavescens Injection with cisplatin for intraperitoneal chemotherapy in patients with gastric cancer and malignant ascites showed improvements in patients’ quality of life and immune function. This was evidenced by significant increases in CD3+, CD4+, CD4+/CD8+ T cell subsets, and NK cell levels, suggesting an enhanced anti-tumor response from CD8+ T cells. Another study (128) revealed that patients treated with ginseng-containing traditional medicine preparations (G-TMPs) alongside fluoropyrimidine-based chemotherapy (FBC) not only experienced improved tumor response and disease control but also exhibited elevated levels of CD3+ and CD4+ T cells, including an improved CD4+/CD8+ T-cell ratio. This enhancement in anti-tumor capability of CD8+ T cells was accompanied by a reduction in the incidence of adverse drug reactions, indicating that G-TMPs may enhance the efficacy and safety of FBC in treating advanced gastric cancer.

##### 3.4.1.2 Enhancing the quantity and activity of NK cells

Research highlights that certain compounds, active ingredients, and external treatments in TCM can significantly enhance both the quantity and activity of NK cells, thereby amplifying their anti-tumor effects. For example, a study explored the synergistic anti-tumor effects of Ganoderma lucidum polysaccharide (GLP) combined with 5-Fu on gastric cancer (129). The findings revealed that GLP substantially boosts the cytotoxic activity of NK cells, particularly NK-92 cells, against gastric cancer cell lines BGC823 and SGC7901. GLP was found to activate the NK cell receptor NKG2D and its downstream DAP10/PI3K/ERK signaling pathway, enhancing the killing effect of NK-92 cells and suggesting a potential therapeutic strategy that combines immunotherapy with chemotherapy for gastric cancer. Additionally, lentinan, an active component extracted from high-quality shiitake mushrooms, has demonstrated remarkable immunomodulatory effects (130). In clinical settings, lentinan has been observed to significantly increase both the count and activity of NK cells in gastric cancer patients, enhancing their capacity to destroy tumor cells and reducing the immunosuppressive effects of chemotherapy.

##### 3.4.1.3 Enhancing the anti-tumor activity and antigen-presenting capability of dendritic cells

DC-based vaccines are being developed to enhance the antitumor immune response by loading DCs with tumor antigens and reintroducing them into the patient to activate cytotoxic T cells (131). In the realm of TCM, Chinese herbal polysaccharides are recognized as “adjuvants” that facilitate DC maturation *in vitro* and enhance the efficacy of DC vaccines. Empirical studies have highlighted the potential of Astragalus to promote DC maturation, likely through the modulation of TLR4-mediated signaling pathways (132). Additionally, Zhou and colleagues (133) investigated the effects of matrine on DCs, including their maturation, cytokine secretion, and the cytotoxic T lymphocyte (CTL) response against gastric cancer. Their findings indicated that matrine dose-dependently enhances the expression of CD86 and CD83, markers of DC maturation. Furthermore, DC vaccines co-treated with LPS and matrine exhibited significantly better CTL killing effects on MKN45 gastric cancer cells than those treated with LPS alone. These results suggest that matrine acts as an effective immunological adjuvant, enhancing DC activation and potentially increasing the effectiveness of autologous DC-based vaccines against gastric cancer.

#### 3.4.2 Reducing immunosuppression and preventing immune escape

##### 3.4.2.1 Reducing the expression and immunosuppressive effect of regulatory T cells

A study by Xu et al. (134) demonstrated that Modified Buzhong Yiqi Tang significantly prolonged the survival of mice bearing gastric cancer, increased the CD4+/CD8+ ratio in the observation group, and decreased the ratio of CD8+ PD-1 T cells and Treg PD-1 cells. Further analysis revealed that this formulation inhibits the expression of PD-L1 in gastric cancer tissues via the PI3K/AKT pathway, effectively suppressing tumor immune escape. Additionally, research on Banxia Xiexin Decoction (BXXX) and its main components, such as Pinellia ternata, Coptis chinensis, and Scutellaria baicalensis, shows that they target key molecules in the PD-L1 pathway, thereby inhibiting cell proliferation and promoting apoptosis in gastric cancer cells. The study highlights that interactions between PD-L1 and PD-1 can inhibit cytotoxic T cell activity and induce the generation of Tregs, which contribute to immune suppression. By inhibiting PD-L1 expression, BXXX may help reduce the immunosuppressive effects of Tregs, thereby enhancing the body’s immune response against gastric cancer (135).

##### 3.4.2.2 Reversing the immunosuppressive phenotype of tumor-associated macrophages

A study examining the efficacy of mJPYZ in treating advanced gastric cancer showed its significant impact on TAMs. The findings revealed that mJPYZ halted gastric cancer progression by decreasing PI3Kγ activity within TAMs, reducing anti-inflammatory IL-10 levels, and increasing pro-inflammatory cytokines such as TNF-α and IL-1β. This shift from an M2 to an M1 phenotype among TAMs contributed to the inhibition of tumor growth and metastasis through the PI3Kγ signaling pathway (136). Similarly, Wu and colleagues (137) demonstrated that the Jianpi Yangzheng Xiaozheng (JPYZXZ) formula was more effective than its individual components at reducing tumor size in tumor-bearing mice. It achieved this by suppressing TAM numbers, reversing M2 differentiation, and enhancing M1 expression in gastric cancer tissues, thereby impeding tumor growth. Additionally, Dendrobium officinale polysaccharide (DOP) has been shown to effectively polarize THP-1 macrophages from the M2 to the M1 phenotype and downregulate the STAT6/PPAR-γ signaling pathway, underscoring its potential as an immunoregulatory agent in gastric cancer treatment and offering a promising approach for managing the tumor microenvironment (138).

##### 3.4.2.3 Inhibiting the negative immune regulation by myeloid-derived suppressor cells

Recent studies have highlighted the efficacy of combining TCM with conventional chemotherapy in treating advanced gastric cancer. For example, Curcumin has demonstrated a significant ability to inhibit tumor growth and tumorigenicity in gastric and colon cancer models by modulating the dynamics of MDSCs. Curcumin treatment reduced the percentages of MDSCs in the spleen, blood, and tumor tissues, decreased IL-6 levels in serum and tumor environments, and inhibited MDSC proliferation. It also suppressed the activation of STAT3 and NF-κB signaling pathways, and promoted a shift in MDSCs towards an M1-like phenotype, highlighting its potential therapeutic benefits (139). Additionally, the JPYZXZ decoction has been shown to inhibit the expression of exosomal PD-L1 derived from gastric cancer cells. This inhibition impacts the expansion and differentiation of MDSCs mediated by these exosomes, alleviating gastric cancer progression in murine models and human patients by disrupting the delivery of exosomal PD-L1 to bone marrow cells (140). Another study focused on the combined effects of Trametes robiniophila Murr (TRM) and 5-FU on gastric cancer. This combination significantly enhanced the anticancer activity of 5-FU, prolonged survival in mice with xenograft tumors, and reduced the risk of liver metastasis. It also favorably altered immune dynamics within the tumor microenvironment by decreasing levels of IL-6, IL-10, and TGF-β, as well as polymorphonuclear MDSCs (PMN-MDSCs), while increasing IFN-γ and NK cells (141).

In summary, TCM has the potential to reshape the immune microenvironment by enhancing the killing capacity of immune cells and negatively regulating the function and activity of immunosuppressive cells, thereby inhibiting tumor growth, recurrence, and metastasis. However, the existing body of research has several limitations, including the complex composition of TCM compounds, multiple targets of action, and challenges in elucidating effective mechanisms, compounded by a scarcity of high-quality clinical studies. Therefore, further exploration of TCM’s regulatory effects on the immune microenvironment through diverse approaches such as transcriptomics, proteomics, and metabolomics, alongside conducting multi-center, large-scale clinical trials, is essential to substantiate the anti-tumor properties of TCM.

## 4 Clinical implications and future directions

TCM offers a promising complementary approach to gastric cancer treatment, characterized by its holistic and multi-targeted strategies. Despite significant advancements in conventional therapies, challenges such as drug resistance, metastasis, and recurrence remain prevalent. TCM’s ability to influence various biological processes involved in cancer progression presents opportunities to address these challenges effectively.

### 4.1 Potential for clinical applications in immunotherapy

The modulation of the immune microenvironment by TCM presents significant opportunities for its integration with immunotherapy—a rapidly evolving area in gastric cancer treatment. TCM formulations that increase CD8^+^ T cell activity and natural killer (NK) cell function may enhance the body’s immune response against tumors, potentially improving the outcomes of immune checkpoint blockade therapies. Additionally, TCM’s ability to downregulate immunosuppressive molecules like PD-L1 could complement the action of PD-1/PD-L1 inhibitors, addressing immune escape mechanisms employed by cancer cells. By enhancing the effectiveness of immunotherapies, TCM could contribute to improved survival rates and quality of life for patients with advanced gastric cancer.

### 4.2 Acknowledging limitations and challenges

Despite these promising findings, several limitations and challenges must be acknowledged. Many studies reviewed are preclinical, involving *in vitro* experiments or animal models, and their results may not directly translate to human physiology due to differences in metabolism and tumor microenvironment. The complexity and variability of TCM formulations pose challenges in standardization and quality control, affecting the reproducibility and reliability of results. Clinical studies on TCM often lack rigorous design, with small sample sizes and inadequate control groups, reducing the strength of evidence. Potential interactions between TCM compounds and conventional drugs are not well understood, raising concerns about safety and efficacy when used concurrently. Additionally, the lack of standardized guidelines and stringent regulatory frameworks limits the integration of TCM into mainstream clinical practice.

### 4.3 Future research directions

To overcome these challenges and fully realize the potential of TCM in gastric cancer treatment, future research should focus on several key areas. Conducting rigorous clinical trials is paramount; large-scale, multicenter randomized controlled trials with robust methodology are necessary to provide definitive evidence of efficacy and safety. Exploring the synergistic effects of TCM when combined with conventional treatments can determine optimal protocols and enhance therapeutic outcomes. Efforts should be made to standardize TCM formulations and implement rigorous quality control measures to ensure the purity, potency, and safety of TCM products, which is crucial for reproducibility and regulatory approval.

Herbal compounds that inhibit key glycolytic enzymes may improve the effectiveness of chemotherapy by reducing the energy supply to cancer cells and sensitizing them to chemotherapeutic drugs. The anti-angiogenic effects of Chinese medicines may limit tumor growth and metastasis, making tumors more sensitive to treatments such as radiotherapy. In addition, the immunomodulatory properties of Chinese medicines, such as enhancing the activity of effector immune cells and reducing the population of immunosuppressive cells, may enhance the efficacy of immunotherapy. This is particularly important given the growing importance of immune checkpoint inhibitors in gastric cancer treatment.

Pharmacokinetic and pharmacodynamic studies are essential to understand how TCM compounds are absorbed, distributed, metabolized, and excreted in the human body. This knowledge can inform optimal dosing strategies, enhance bioavailability, and reduce potential toxicity. Research into novel delivery systems or chemical modifications could improve the absorption and effectiveness of TCM compounds. Additionally, exploring personalized medicine approaches by identifying genetic or molecular biomarkers that predict patient response to TCM treatments can facilitate tailored therapy, improving efficacy and minimizing adverse effects. Integrating omics technologies such as genomics, proteomics, and metabolomics can help understand individual variations in response to TCM and guide personalized treatment strategies.

Addressing potential herb-drug interactions is crucial for patient safety. Systematic studies are needed to evaluate these interactions, and developing evidence-based guidelines on the co-administration of TCM and standard treatments can aid clinicians in making informed decisions. Collaborating with regulatory bodies to create clear guidelines for the approval and use of TCM products can facilitate their integration into clinical practice. International cooperation to harmonize standards for TCM research and practice will enhance global acceptance and utilization.

## 5 Conclusions

Gastric cancer remains a significant global health challenge, particularly due to metastasis and drug resistance in advanced stages. TCM offers a promising complementary approach with its holistic and multi-targeted strategies. This review highlights how TCM inhibits key processes in gastric cancer progression: (1) Inhibiting Aerobic Glycolysis: TCM reduces the energy supply essential for cancer cell proliferation by downregulating key glycolytic enzymes and metabolic pathways. (2) Suppressing Angiogenesis: By targeting the VEGF and COX-2 pathways, TCM effectively hinders the formation of new blood vessels necessary for tumor growth and metastasis. (3) Modulating the Immune Microenvironment: TCM enhances the activity of immune cells like CD4^+^ and CD8^+^ T cells and NK cells while reducing immunosuppressive cells such as Tregs and MDSCs, strengthening the body’s antitumor response. Despite these promising mechanisms, challenges remain in translating TCM findings into clinical practice due to issues like standardization, quality control, and limited clinical trials. Addressing these challenges through rigorous research and integration with conventional therapies could enhance the efficacy of gastric cancer treatment.

In summary, TCM holds significant potential in improving gastric cancer outcomes by targeting multiple pathways involved in tumor progression. Its integration into comprehensive treatment strategies may offer better therapeutic efficacy and improved quality of life for patients.
